# Carbohydrate Supplementation Does Not Improve 10 km Swimming Intermittent Training

**DOI:** 10.3390/sports6040147

**Published:** 2018-11-14

**Authors:** Roberto Baldassarre, Massimo Sacchetti, Federica Patrizio, Andrea Nicolò, Alessandro Scotto di Palumbo, Marco Bonifazi, Maria Francesca Piacentini

**Affiliations:** 1 Department of Movement, Human and Health Sciences, University of Rome Foro Italico, 00135 Rome, Italy; roberto.baldassarre@me.com (R.B.); massimo.sacchetti@uniroma4.it (M.S.); federica.patrizio@uniroma4.it (F.P.); andrea.nicolo@yahoo.com (A.N.); a.scottodipalumbo@hotmail.it (A.S.d.P.); 2 Department of Medical, Surgical and Neuro Sciences, University of Siena, 53100 Siena, Italy; marco.bonifazi@unisi.it

**Keywords:** endurance, performance, nutrition

## Abstract

The aim of the present study was to test the effectiveness of carbohydrate (CHO) feeding supplemented every 2.5-km, as in official races, on the performance, rating of perceived exertion (RPE), and glycaemia during a 10-km intermittent training workout in elite open-water swimmers. A randomized crossover design was used. Participants completed two 10-km intermittent training sessions (20 × 500-m). The relative velocity was expressed in percentage of a single 500-m. Glycaemia was monitored by continuous glucose monitoring. Participants had to ingest either 1 L of tap water (WAT; 0.50 L·h^−1^) or 120 g of CHO in the form of 8% solution (60 g·h^−1^). The 15-point RPE scale was used during the trials. A two-way ANOVA for repeated measures was performed (*p* < 0.05). The relative velocity of each 500-m was not significantly different between the two trials. No significant differences emerged in the relative velocity of the last 500-m between trials. Average RPE was not statistically different between the two trials (11 ± 3 in WAT and 12 ± 3 in CHO). In the last 500-m, glycaemia was significantly higher in the CHO trial (5.92 ± 0.47 mmol·L^−1^ in CHO; 5.61 ± 0.61 mmol·L^−1^ in WAT). CHO ingestion did not improve performance or affect RPE during a 10-km intermittent training in elite open-water swimmers.

## 1. Introduction

Competitive swimming includes 17 pool individual events from 50-m to 1500 m (21 s to approximately 15 min) and three open water events: 5–10 and 25-km, lasting from less than 1 h to approximately 6 h. Independently, distance elite swimmers perform approximately 55–80 km per week [1,2,3] and frequently participate in multiple high-volume training sessions per day [3].

Open-water swimming races include three official race distances: 5, 10, and 25-km, but only the 10-km is an Olympic event since 2008 [4]. The 10-km race is an event that lasts around 1 h 50 min for the best male and 1 h 56 min for the best female swimmers. Environmental challenges (unpredictable waves, tides, and currents) may have an influence on the effective distance covered by swimmers and also on their nutritional strategies [5]. Currently, both the shorter (5–10-km) and the longer (25-km) open-water events are completed on a loop course (normally of 2.5-km), and at the end of each loop floating or stationary feeding stations are positioned. Accredited handlers are allowed to pass solids and liquids through long poles with a cup or delivery vessel attached [6]. However, contrary to running, feeding for swimmers is unpractical. First, the feeding stations are positioned off the race line, therefore, swimmers are forced to deviate from the optimal line and from drafting; and secondly, athletes normally roll on their back once they collect their cup, and then roll back over and this might bring about a decrease in swimming speed [6]. Because the duration and intensity of a 10-km open water race corresponds to the theoretical limits of glycogen storage [6], athletes are aware of the importance of carbohydrate (CHO) supplementation during the race [7].

The ergogenic effects of CHO feeding during continuous and intermittent endurance exercise have been consistently demonstrated in numerous studies [8,9,10,11,12] and reviews [13,14,15,16,17,18].

During prolonged strenuous endurance tasks, there is a drop in the total carbohydrate oxidation rate, depletion in skeletal muscle glycogen, and a decline in blood-glucose availability to the contracting muscle, probably secondary to liver glycogen depletion [15]. Specifically, the high volume training adopted by elite swimmers can significantly deplete muscle glycogen stores [19], and carbohydrate intake during a workout can provide additional fuel to support performance in a particular session [6,20].

Ingestion of carbohydrates during prolonged endurance exercise produces several beneficial effects, such as an attenuation in central fatigue, a better maintenance of CHO oxidation rates, muscle glycogen sparing, changes in muscle metabolite levels, a reduction of stress hormones and inflammatory cytokines, and a better maintenance of excitation-contraction coupling [13]. Costill et al. [19] demonstrated that swimmers who did not increase carbohydrate intake in response to an increased training volume were unable to complete efficiently the programmed training compared to swimmers who increased carbohydrate intake to maintain muscle glycogen stores.

The ergogenic effect of glucose supplementation is often ascribed to a higher glucose uptake by the exercising muscles, thereby allowing a sufficient carbohydrate oxidation late in exercise when muscle glycogen levels are low [9,11]. Moreover, CHO ingestion has been shown to reduce the rating of perceived exertion (RPE) during continuous endurance exercise [8,21,22,23].

However, the current nutritional guidelines utilized by open-water swimmers are extrapolated from other sports with a similar duration and physiological requirements [5,6,24]. In fact, the vast majority of studies have been performed with endurance athletes (runners or cyclists) performing continuous exercise, and very little research is available regarding nutritional practices [24] and fuel utilization [18] during training and competition in highly trained athletes and, specifically, open-water swimmers [5].

Therefore, the aim of the present study was to test the effectiveness of CHO feeding supplemented every 2.5-km, as in official races, on performance, RPE, and glycaemia during a 10-km intermittent training workout in elite open-water athletes. It was hypothesized that CHO supplementation guidelines, extrapolated from other sports with a similar duration, may be suitable for swimmers, with the main effect of improving performance and reducing RPE.

## 2. Methods

### 2.1. Participants

Ten elite open-water swimmers (6 males and 4 females) volunteered to take part in this study. Volunteers mean age, height, and weight were 22 ± 5 years (23.5 ± 5 males and 21 ± 2 females), 1.76 ± 0.05 m (1.78 ± 0.04 males and 1.73 ± 0.06 females), 70.5 ± 7 kg (72.50 ± 6 males and 65.33 ± 6 females) respectively. The inclusion criteria required participants to have performed at least one international open-water competition (World Cup, European championships, World championships, or Olympic Games), the ability to swim 5000-m in a 50 m swimming pool within 55 and 60 min, respectively, for male and female participants, and no history of metabolic diseases.

Participants were informed about the purpose and procedures of the study, which was approved by the local ethical committee (code CARD2018/10) in accordance with the Declaration of Helsinki.

All athletes provided written consent before participation.

### 2.2. Experimental Design

Participants completed two main trials, each separated by 2 days of rest, and the order was randomized to counteract order effects. Both trials were conducted under similar environmental conditions, in a 25-m indoor swimming pool, with a water temperature of 27 °C.

The day before the first trial, a continuous glucose monitoring system (CGMS) was applied on the left side of the lower back, allowing a correct calibration of the system.

Participants were asked to consume a high-carbohydrate diet (8–10 g·kg^−1^ per day [6,25]) during the 2 days prior to the first trial as they would normally do before a race. Food and drink intake were recorded by a personal diary and the diet was replicated before the second trial.

Athletes arrived in the swimming pool at 8.00 a.m., following an 8–9 h sleep. Each participant consumed his or her regular pre-race breakfast at least 2 h before the trial. After a standard warm up (30-min), athletes performed a 10-km intermittent training session, divided in 20 sets of 500-m. This is a typical session utilized by the coach, several times during a season, to monitor training progression; in fact, although elite open-water swimmers perform the majority of their training below the first ventilatory threshold, about 23% is performed at higher intensities [1]. Therefore, athletes were all familiarized with this type of training. The participants were divided in four time-groups, with different fixed restart times in each set (5:50; 6:00; 6:15; 6:30 min:s). The coach defined the rest time through a single 500-m (500_max_) performed 1 month prior to the first trial. Participants were instructed to perform the first 9.5-km of training at an intensity between 80% and 90% of their 500_max_ and the last 500-m as fast as possible, replicating a typical open-water swimming race strategy [26,27]. During both sessions, verbal encouragements, technical recommendations, and feedback on split times were given by the coach to all athletes. Each athlete swam in his or her own lane and next to another athlete within the same time-group. In order to evaluate the performance and reduce the velocity variability in the female and male participants, the relative velocity was expressed as a percentage of personal 500_max_ (%-500_max_).

On each occasion, participants were asked to ingest either tap water (WAT) or a solution of water plus carbohydrate (CHO). During the trials, swimmers drank three times every 2.5-km in both conditions in order to simulate feeding zones during the race. In the WAT trial, the swimmers ingested approximately 1.5 L of tap water, ~0.47 L each time (~0.50 L·h^−1^), as indicated by the guidelines to prevent dehydration status [28]. In the CHO trial, the swimmers ingested 120 g of carbohydrate in the form of 8% solution (glucose:fructose ratio of 1:1; Enervitene Sport Cheerpack, Enervit©, Milan, Italy) with an ingestion rate of 60 g·h^−1^ (~0.47 l of water plus 40 g of carbohydrate each time), as indicated by guidelines for endurance exercise of the same time duration [6,14,15].

The 15-point Rating of Perceived Exertion Scale (RPE) [29] was used to assess perceived exertion during the trials. The scale ranges from 6 to 20, with verbal-anchors ranging from ‘‘no effort” to ‘‘maximum effort”. RPE was administered every 1000-m and after training through a clearly visible poster. All athletes had been familiarized with the RPE scale before the first trial.

### 2.3. Continuous Glucose Monitoring

Glycaemia was monitored by a Continuous Glucose Monitoring System (CGMS^®^ iPRO TM—Medtronic©, Northridge, CA, USA). Participants were instructed on the use of the device and were asked to measure capillary blood glucose four times daily using a personal glucometer (Contour^®^ Next Link, Bayer, Germany). These measurements were used to calibrate the sensor. Interstitial glucose was continuously measured in the subcutaneous tissue every 5 min during the trials.

The accuracy and validity of the CGMS during exercise was assessed in previous studies [30,31], and has been shown to be waterproof.

The device was applied on the left side of the lower back, in order to allow all swimmers the possibility to do their normal freestyle flip turn during both trials.

### 2.4. Statistical Analysis

Data are presented as mean ± standard deviation (SD). The confidence interval at 95% of the difference of the means (95% CI), Hedge’s G effect size (g), and observed power were indicated when appropriate. All statistical analysis was performed using the statistical software, PASW statistics 22 (SPSS Inc., Chicago, IL, USA). All data were tested for normal distribution using a Shapiro-Wilk test and the sphericity was checked with the Mauchly’s test. A two-way ANOVA for repeated measures was used to assess the different effects of CHO and WAT on RPE, glycaemia, and velocity. When a significant F-value was achieved, Bonferroni adjustment procedure was performed to locate the pairwise differences. Where appropriate, comparison of variables between two conditions was conducted by using a Student’s *t*-test for paired samples. The level of significance was set at *p* ≤ 0.05.

## 3. Results

### 3.1. Performance

**Figure 1:**
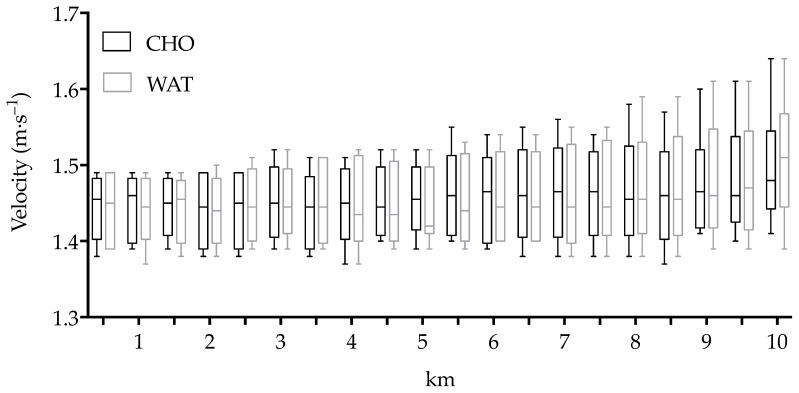
Median and interquartile ranges of the velocity (m.s^−1^) maintained during each km in the CHO and WAT trials. Boxplot lower and upper whiskers represent the minimum and maximum value, respectively.

The absolute and relative velocity expressed as a percentage of 500_max_ is reported in Table 1. No significant differences emerged in the relative velocity between the trials (F(1,9) = 0.002, *p* = 0.963, 95% CI [−0.626, 0.601], observed power 0.050, g = 0.019; Table 1). No order effect was evident between the first and second trial (F(1,9) = 0.511, *p* = 0.493, 95% CI [−0.408, 0.785], observed power 0.511, g = 0.180). Pacing was not different between the two trials (F(1,9) = 0.000, *p* = 1.00; Figure 1). The relative velocity from the first to the last 500-m (t(9) = −0.579, *p* = 0.577, CI [−5.082, 3.010]; 3.59 ± 3.41% in CHO and 4.63 ± 4.08% in WAT) and from the 19th to the last 500-m (*p* = 1.000; 0.86 ± 1.76% in CHO and 1.80 ± 2.37% in WAT) were not significantly different between the two trials. No significant differences emerged in the relative velocity of the last 500-m between the two trials.

### 3.2. Rating of Perceived Exertion

RPE did not show a significant main effect between the two conditions (F(1,9) = 1.922, *p* = 0.199, 95% CI [−1.158, 0.278], observed power 0.237, g = −0.333), and order effect between the first and second trial (F(1,9) = 4.378, *p* = 0.066, 95% CI [−0.049, 1.249], observed power 0.464, g = 0.319).

The average RPE was 11 ± 3 and 12 ± 3 in the CHO and WAT trials, respectively (Table 1).

**Figure 2:**
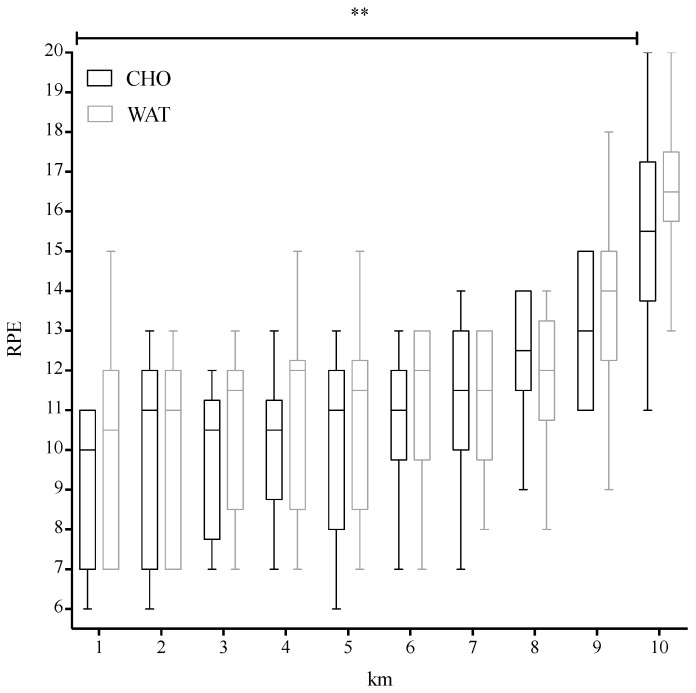
Median and interquartile ranges of RPE reported during each km in the CHO and WAT trials. Boxplot lower and upper whiskers represent the minimum and maximum value, respectively. ** *p* < 0.001.

A significant main effect was observed in the time course in both conditions (F(9,81) = 36.158, *p* < 0.001, observed power 1.00; Figure 2); RPE increased significantly over time across both conditions with the highest value observed at the end of the exercise.

The percentage increment of RPE from the first to the last 500-m was not significantly different between the two trials (t(11) = −0.469, *p* = 0.579, 95% CI [−3.010, 5.082]; 70.27 ± 37.01% in CHO and 74.99 ± 47.61%).

### 3.3. Glucose Concentration

Glycaemia did not show a significant main effect between the two conditions (F(1,9) = 1.445, *p* = 0.260, 95% CI [−2.313, 7.561], observed power 0.190, g = 0.250); however, there was a significant main effect in time course in both conditions (F(24,216) = 44.89, *p* < 0.001, observed power 1.00) and a significant interaction effect between conditions and time course (F(24,216) = 5.71, *p* < 0.001, observed power 1.00). The order effect was not significant between the first and second trial (F(1,9) = 0.753, *p* = 0.408, 95% CI [−7.070, 3.150], observed power 0.122 g = 0.346).

**Figure 3:**
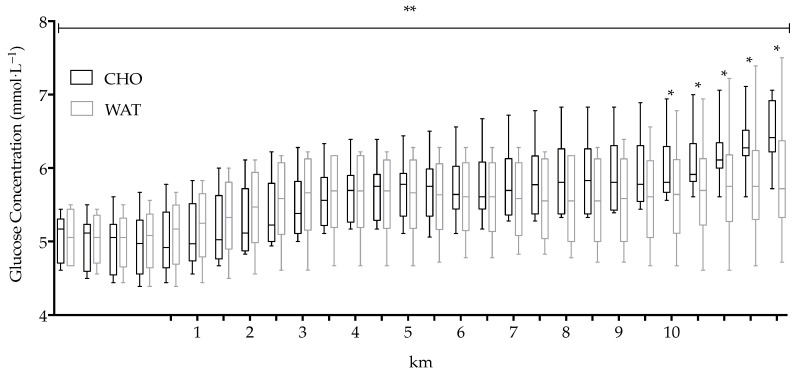
Median and interquartile ranges of glucose concentration (mmol·L^−1^) during each km in the CHO and WAT trials. Boxplot lower and upper whiskers represent the minimum and maximum value, respectively. * *p* < 0.05; ** *p* < 0.001.

Glycaemia was not different between trials at the beginning of exercise (4.97 ± 0.40 mmol·L^−1^ in CHO; 5.04 ± 0.39 mmol·L^−1^ in WAT; Figure 3), and remained similar between both trials until km 4.5; thereafter it increased in the CHO trial only (Figure 3), reaching significance only at 9.5-km (5.92 ± 0.47 mmol·L^−1^ in CHO; 5.61 ± 0.61 mmol·L^−1^ in WAT; t(9) = 2.285, *p* = 0.048, 95% CI [0.568, 11.143]; Figure 3). Average glycaemia during exercise was higher in CHO (5.62 ± 0.51 mmol·L^−1^) compared to the WAT trial (5.53 ± 0.54 mmol·L^−1^; Table 1). The percentage increment of glycaemia from the first to the last 500-m was significantly different between the two trials (t(9) = 2.772, *p* = 0.022, CI [1.608, 15.887]; 19.39 ± 4.29% in CHO and 10.64 ± 7.75% in WAT).

Immediately post-exercise, glycaemia continued to increase in the CHO trial compared with the WAT trial. Significant differences were noted 5, 10, 15, and 20 min post-exercise between the two trials (t(9) = 2.867, *p* = 0.019, 95% CI [1.350, 11.449]; t(9) = 2.985, *p* = 0.015, 95% CI [1.768, 12.831]; t(9) = 2.967, *p* = 0.016, 95% CI [2.138, 15.861] and t(9) = 2.643, *p* = 0.027, 95% CI [1.570, 20.229], respectively; Figure 3).

## 4. Discussion

Because the duration and intensity of a 10-km open-water race corresponds to the theoretical limits of glycogen storage [6], the primary aim of the present study was to evaluate the effects of 60 g·h^−1^ of CHO during a 10-km intermittent training workout in elite open-water athletes. Swimmers are in fact encouraged to follow these nutritional guidelines, however, CHO ingestion did not improve performance or reduce RPE during a 10-km intermittent training.

In the present study, the athletes were asked to adopt the typical strategy they would adopt during a 10-km race: An initial even pace lower than the average race pace and an increase in speed in the last km [9,27]. The relative velocity from the first to the last set and from the 19th to the last were not significantly different between the two trials. Furthermore, no significant differences emerged in the relative velocity of the last 500-m between trials (Figure 1).

Green et al. [32] suggest that it is reasonable to assume that pacing strategies improve through repetition. Years of high-level training may produce several chronic adaptations in elite open-water athletes that allow them to select the more suitable pacing strategies and to sustain two 10-km training bouts with a short recovery time and with an identical pacing strategy. These data confirm previous results on the pacing ability of elite athletes that are able to self-select and maintain an even pace during exercise compared to novice athletes [32].

It is scientifically proven that CHO beverage supplementation increases performance both during continuous [8,9,10,11] and intermittent [12] endurance exercise. Recently, a systematic review [16] reported that 83% of selected studies showed statistically significant benefits of CHO intake compared to placebo in exercise bouts between 1 and 2 h. The mental-cognitive stimulation of the central nervous system and the increase of carbohydrate oxidation rate with concurrent decrease of glycogen utilization are the major mechanisms that explain why carbohydrate supplementation during exercise improves performance [10,11,16]. However, the same review reported that 18% of selected studies showed no change in performance after the intake of carbohydrates compared with placebo [16]. Madsen et al. [33] showed that the ingestion of a 5% carbohydrate solution (66 g·h^−1^) during 100-km of cycling did not enhance performance in well-trained cyclists. Similar results were obtained by McConell et al. [34] in a group of 13 well-trained males cyclists and triathletes that consumed a 6% carbohydrate solution (81 g·h^−1^). In this case, glucose ingestion increased glucose uptake and partially reduced endogenous glucose production, but had no effect on carbohydrate oxidation, muscle metabolism, and did not increase time to exhaustion of exercise [34].

The type or blend of CHO can affect the effects of supplementation on performance [14,16]. We opted for a glucose:fructose ratio of 1:1 according to the guidelines for endurance exercises of the same time duration [6,14,15]. These guidelines are based on laboratory studies that used cycling or running exercises, while the validity of CHO intake recommendations for swimming for 2 h remains to be confirmed [6,24].

According to Shaw et al. [6] open-water events longer than 5-km (typically, the 10 and 25-km) might increase the glycogen depletion rate. Costill et al. [19] showed that a 5-km interval training depleted muscle glycogen stores in male collegiate swimmers, and was associated with a decline in the distance per stroke at a given speed. The majority of propulsive forces in swimming is gained from the use of the arms [35]. The different muscle masses involved in swimming, compared with cycling or running, suggests a different energy metabolism during exercise. In fact, the different hemodynamics of the active muscle mass and the greater reliance on fast twitch fibres during arm exercise implies a different substrate oxidation rate compared to leg exercise [36]. When exercise is performed at the same relative load, CHO oxidation is higher and, subsequently, fat oxidation is lower during arm compared to leg exercise [36].

The supplementation strategies for upper body exercises are underreported in the literature and few studies have been conducted during endurance exercise of more than 60 min. Tremblay et al. [37] compared fuel selection during prolonged moderate arm and leg exercise (120 min at 50% maximal aerobic power output), with and without glucose ingestion in a group of active male participants. The energy yield from fat oxidation was significantly lower when exercising with the arms compared to the legs in both conditions (water and glucose), and was significantly decreased with glucose ingestion in arm, but not leg, exercises. The energy yield from CHO oxidation was not significantly different between arms and legs when water was ingested, while it significantly increased when CHO was ingested in arms exercise only. Moreover, the exogenous CHO oxidation was significantly higher during arm compared to leg exercise while the endogenous CHO oxidation significantly decreased with glucose ingestion and was not significantly different in the two modes of exercise. The difference in fuel selection observed between the two modes of exercise when water was ingested was modest, with a slightly higher reliance on CHO oxidation during arm compared to leg exercise in active male participants [37].

However, the training level of participants may affect substrate utilization during exercise. Hetlelid et al. [38] showed that well-trained (70 ± 5 mL·Kg^−1^·min^−1^ of VO_2peak_) and recreational (55 ± 5 mL·Kg^−1^·min^−1^ of VO_2peak_) runners performed a high-intensity intermittent exercise (6 × 4 min of work separated by 2 min of rest) at similar levels of RPE, blood lactate accumulation, and at comparable CHO oxidation rates, while fat oxidation was 0.66 and 0.26 g·min^−1^ in well-trained and recreational runners, respectively. Trained athletes are able to oxidize more fat sparing muscle glycogen with the same exogenous CHO oxidation rate during prolonged moderate-intensity exercise compared to untrained participants [39]. The capacity to oxidise fat at high exercise intensities is a supremely advantageous adaptation for endurance athletes [38]. Years of high-level training have been shown to determine several adaptations (such as increased maximal oxygen uptake, oxidative enzyme activity, peripheral capillarization, mitochondrial density) that improve the fat oxidation rate during endurance exercise. It can be hypothesized that elite open-water swimmers have a high ability to oxidise fat with a consequent sparing of endogenous CHO, reducing the effect of CHO supplementation exercise with predominant use of the arms, specifically if reproducing intensities normally observed in race settings (first part of the race at low intensities and final end spurt) [27].

Furthermore, the rate of carbohydrate oxidation depends on diet and glycogen levels [40]). Our participants were in a fed state and were asked to replicate the same high-carbohydrate diet before each trial to maximize the glycogen storage. Moreover, sufficient time (two days) was granted to restore glycogen storage between the trials [25]. The liver and muscle glycogen stores are important to prevent hypoglycaemia during exercise and fasting. When liver and muscle glycogen stores are depleted, hypoglycaemia may occur, but no participant suffered from hypoglycaemia (<3.9 mmol·L^−1^) in both trials.

Additionally, the intensity of exercise clearly affects energy substrate utilization [40], and plasma glucose levels (glycaemia) increase proportionally to exercise intensity. In the current study, glycaemia increased in the CHO trial with no difference observed in the WAT, despite the pacing and relative velocity from the first to the last 500-m being not different between the two trials. Moreover, immediately post-exercise, glycaemia levels continued to increase. The glycaemia values obtained in the present study were comparable to other studies [8,10,12], however, the different protocols of exercise and level of athletes selected did not allow a complete comparison between studies.

The second finding of this study was that carbohydrate ingestion did not reduce RPE during intermittent training. Conversely, CHO ingestion during exercise has been shown to attenuate RPE and reduce the feeling of effort during continuous exercise protocols in cycling [8] and running [21,23]. Utter et al. [41] observed an attenuation of RPE during prolonged cycling intermittent exercise (120-min), when a 6% CHO solution was consumed compared to a water placebo. Central fatigue seems to be counteracted when blood glucose homeostasis is maintained, and it is easier for the participants to retain power output at the end of a prolonged exercise bout when hypoglycaemia is prevented via carbohydrate supplementation [9]. Lower RPE was associated with a higher level of carbohydrate oxidation, higher plasma glucose and insulin levels, and lower plasma cortisol and growth hormone levels [22]. Since RPE is a strong predictor of exercise tolerance [42], a decrease of RPE (after CHO ingestion) may improve performance.

In the present study, no statistical differences were observed in RPE between trials. Although RPE increased significantly over time across both conditions, the percentage increment of RPE from the first to last set was not significantly different between the two trials.

The average RPE was 11 “light”, which may suggest that participants swam at a relatively low intensity for 2 h without depleting glycogen, although the relative average velocity was approximately 87% compared to 500_max_ (Table 1). Furthermore, the time to complete both trials (without considering rest time) was similar to the 10-km seasonal best time achieved by the athletes in the same season. Although the difference in relative velocity during the last 500-m between CHO and WAT trials was not significant (0.99 ± 4.87%), during the Rio Olympic Games (2016), the performance difference between the first and the 10th finisher was 0.07% in males and 0.81% in females [5]. The great variability observed in the last 500-m between CHO and WAT trials suggested that each swimmer must select the best nutritional strategy based on individual physiological characteristics. We do believe that a variation of ~1% in the last set could mean winning or losing a medal also if statistically not significant. Therefore, ingestion of 60 g·h^−1^ of CHO may have a positive effect during the final phases of a 10-km swimming performance.

A limitation of the present experimental design was the absence of a control trial with an artificially non-caloric sweetened placebo and a limited number of participants. However, the inclusion of only elite athletes reduced the possibility of recruitment. A double-blind, placebo-controlled study is needed to definitively clarify the effect of CHO supplementation during endurance swimming exercise. However, we believe that there was not a “placebo” effect considering that we did not find any increase in performance in the CHO trial. The placebo effect is a favorable outcome arising purely from the belief that one has received a beneficial treatment [43]. Furthermore, Hulston and Jeukendrup [44] showed that there was no placebo effect when participants believed they had ingested carbohydrate compared to water or placebo drink.

Finally, the present study tested the possibility to use a continuous glucose monitoring system during swimming. To our knowledge, there are not studies that have monitored glycaemia levels through a CGMS during swimming exercise, while two previous studies used a CGMS during an ultra-marathon race (100-km) [45,46]. The athletes involved did not report any discomfort during trials or rest days.

## 5. Conclusions

In conclusion, the ingestion of 60 g·h^−1^ of CHO did not significantly improve performance and did not affect RPE during a 10-km intermittent training in elite open-water swimmers. This study represents the first attempt in monitoring continuously glycaemia levels during a 10-km swimming training in elite athletes. Future nutritional studies are needed in laboratory and ecological settings in order to quantify the correct dosage and/or types of CHO supplementation for swimmers to have a performance enhancement in competition and training, respectively. Moreover, the duration of some open-water events involves physiological issues (thermoregulatory challenges, significant fluid loss, muscle fuel depletion) not typically seen in other endurance events [6] that may affect nutritional strategies.

## Figures and Tables

**Table 1:** Velocity data in CHO and WAT trials.

	CHO				WAT				500_max_
	Average	1st	19th	20th	Average	1st	19th	20th	
Velocity (m·s^−1^)	1.46 ± 0.05	1.44 ± 0.04	1.48 ± 0.07	1.50 ± 0.07	1.46 ± 0.06	1.44 ± 0.04	1.48 ± 0.08	1.51 ± 0.08	1.60 ± 0.06
%-500_max_ (%)	87.14 ± 3.33	86.28 ± 2.54	88.64 ± 4.21	89.39 ± 4.32	87.16 ± 3.61	86.20 ± 2.60	88.63 ± 4.72	90.20 ± 4.67	
Males									
Velocity (m·s^−1^)	1.49 ± 0.05	1.47 ± 0.03	1.51 ± 0.08	1.52 ± 0.08	1.50 ± 0.05	1.47 ± 0.03	1.53 ± 0.06	1.56 ± 0.05	1.64 ± 0.04
%-500_max_ (%)	89.01 ± 2.82	87.80 ± 1.56	90.43 ± 4.51	90.76 ± 4.78	89.34 ± 3.02	87.83 ± 1.83	91.60 ± 3.61	93.20± 3.15	
Females									
Velocity (m·s^−1^)	1.41 ± 0.03	1.41 ± 0.03	1.44 ± 0.03	1.46 ± 0.05	1.40 ± 0.02	1.40 ± 0.02	1.41 ± 0.01	1.43 ± 0.03	1.55 ± 0.03
%-500_max_ (%)	84.35 ± 1.68	84.01 ± 1.95	85.94 ± 1.77	87.35 ± 2.94	83.89 ± 1.06	83.76 ± 1.24	84.18 ± 0.88	85.70 ± 1.94	

**500_max_** = seasonal best time on 500-m; **%-500_max_** = velocity expressed as % of 500_max_.
